# Supplementary material for: Colossal tunability in high frequency magnetoelectric voltage tunable inductors
Source: Nat Commun. 2018 Nov 27;9:4998. doi: 10.1038/s41467-018-07371-y (PMC6258707; doi:10.1038/s41467-018-07371-y)
Supplement: Supplementary file 1 — Supplementary Information [file 41467_2018_7371_MOESM1_ESM.pdf]

Colossal tunability in high frequency magnetoelectric voltage tunable inductors (Supplementary information)

Yongke Yan[1,2], Liwei D. Geng[3], Yaohua Tan[4], Jianhua Ma[4], Lujie Zhang[5], Mohan Sanghadasa[6], Khai Ngo[5], Avik W. Ghosh[4], Yu U. Wang[3], and Shashank Priya[1,2]

[1]Center for Energy Harvesting Materials and Systems, Virginia Tech, Blacksburg, VA 24061, USA

[2]Department of Materials Science and Engineering, The Pennsylvania State University, University Park, PA, 16802

[3]Department of Materials Science and Engineering, Michigan Technological University, Houghton, MI 49931, USA

[4]Department of Electrical and Computer Engineering, University of Virginia, Charlottesville, VA 22904, USA

[5]Center for Power Electronics Systems (CPES), Virginia Tech, Blacksburg, VA 24061, USA

[6]Weapons Development and Integration Directorate, Aviation and Missile Research, Development, and Engineering Center, US Army RDECOM, Redstone Arsenal, AL 35898, USA

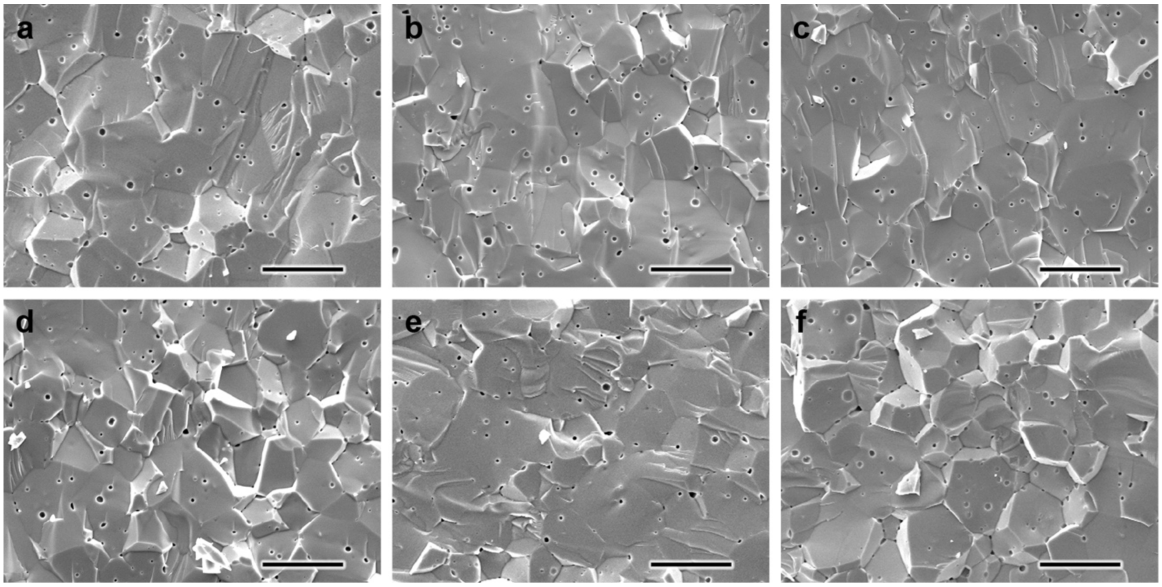

Supplementary Figure 1 | Microstructure of ferrites. SEM images of NZCF-100*x*CFO: (a) x =0, (b) x =0.01, (c) x =0.02, (d) x =0.03, (e) x =0.04, and (f) x =0.05. All samples were sintered at 1050 ^{o}C for 2 hours in air. The microstructures of all NZCF-100*x*CFO ferrite show high density with similar grain size. Scale bars in all images represent 10 μm.

Supplementary Note 1: First Principles Calculations

All calculations presented in this work were based on the projector-augmented wave PAW method which is implemented in the Vienna ab initio simulation package (VASP). The GGA+U with PBE functional were used as it was suggested by Ref.1. A Cut off energy 500 eV and k-space grid of 5×5×3 are used for all calculations for good convergence. For GGA+U calculation, we used $U_{\text{eff}}=U-J=3$ eV for Fe, Co and Ni.[1] We use the pseudo-potential contributing 15 valence electrons per Co($3p^6 4s^2 3d^7$), 10 valence electrons per Ni($4s^2 3d^8$), 14 valence electrons per Fe($3p^6 4s^2 3d^6$), and 6 valence electrons per O($2s^2 2p^4$).

CFO and NFO are inverse spinel structure (space group Fd3¯m, general formula AB_2X_4). The trivalent cations occupy all A sites as well as 50% of the B sites whereas the remaining 50% of the B sites are occupied by the divalent cations. In the inverse spinel structure, Fd3¯m symmetry is preserved suggesting that the distribution of divalent and trivalent cations on the B sites is completely random. To enable our calculations for random distribution, we considered a unit cell with twice the size of the primitive unit cell. In particular, unit cells with Imma, P4m2 and $P4_122$ symmetries are considered in the calculation as suggested by Ref. 2. The crystal with Imma symmetry is shown in Fig.3 (b). During the geometric relaxation process, we fixed Co/Ni and Fe site, varies O positions during relaxation.

We first determined the equivalent lattice structure of CFO/NFO by relaxation based on GGA+U within collinear calculations. After that, we determined the CFO/NFO geometries under symmetry breaking strain. Based on the relaxed structures, we performed non-collinear calculations to calculate magnetic anisotropy and magnetostriction.

To calculate magnetic anisotropy and magnetostriction, we model the total energy by following model.

$$\overline{E}\left(\vec{\varepsilon},\vec{\alpha}\right)=f_K+f_{el}+f_{me} \tag{1}$$

$$f_K^{\ cubic}=K_1\left(\alpha_x^2\alpha_y^2+\alpha_x^2\alpha_z^2+\alpha_y^2\alpha_z^2\right)+K_2\alpha_x^2\alpha_y^2\alpha_z^2 \tag{2}$$

$$f_{el}^{cubic} = \frac{1}{2}C_{11}\left(\varepsilon_{xx}{}^2 + \varepsilon_{yy}{}^2 + \varepsilon_{zz}{}^2\right) + \frac{1}{2}C_{44}(\varepsilon_{xy}{}^2 + \varepsilon_{xz}{}^2 + \varepsilon_{yz}{}^2) + C_{12}(\varepsilon_{xx}\varepsilon_{yy} + \varepsilon_{xx}\varepsilon_{zz} + \varepsilon_{yy}\varepsilon_{zz}) \quad (3)$$

$$f_{me}^{cubic} = B_0\left(\varepsilon_{xx} + \varepsilon_{yy} + \varepsilon_{zz}\right) + B_1\left(\alpha_x^2\varepsilon_{xx} + \alpha_y^2\varepsilon_{yy} + \alpha_z^2\varepsilon_{zz}\right) + B_2\left(\alpha_x\alpha_y\varepsilon_{xy} + \alpha_x\alpha_z\varepsilon_{xz} + \alpha_y\alpha_z\varepsilon_{yz}\right) \quad (4)$$

where C_{11}, C_{12}, and C_{44} are elastic constants and B_0, B_1, and B_2 are magnetoelastic coupling constants. To determine the three independent cubic elastic constants C_{11}, C_{12}, and C_{44} and cubic magnetoelastic coupling constants B_1, and B_2 by distorting the equilibrium crystal structure in three different ways: (1) hydrostatic strain which satisfies $\varepsilon_{xx} = \varepsilon_{yy} = \varepsilon_{zz}$ (isotropic volume expansion), (2) biaxial strain $\varepsilon_{xx} = \varepsilon_{yy} = -0.5\varepsilon_{zz}$ and (3) applying a volume-conserving shear strain ε_{ij}. The magnetostriction coefficients for a cubic system are then calculated by:

$$\lambda_\alpha = -\frac{B_0 + B_0/3}{C_{11} + 2C_{12}}, \quad \lambda_{100} = -\frac{2}{3}\frac{B_1}{C_{11} - C_{12}}, \quad \lambda_{111} = -\frac{B_2}{3C_{44}} \quad (5)$$

where λ_α is the pure volume magnetostriction coefficient, λ_{100} and λ_{111} are the magnetostriction coefficients along [100] and [111]. In particular, we choose biaxial strain $\varepsilon_{xx} = \varepsilon_{yy} = -0.5\varepsilon_{zz} = \varepsilon$, and $\vec{\alpha} = [1,0,0]$, $\vec{\alpha} = [0,0,1]$, in order to calculate the magnetostriction λ_{100}. For this kind of strain, the total energies of the systems are written as

$$\begin{aligned} \bar{E}\left(\varepsilon, \alpha = [1,0,0]\right) &= 3\left(C_{11} - C_{12}\right)\varepsilon^2 + B_1\varepsilon \\ \bar{E}\left(\varepsilon, \alpha = [0,0,1]\right) &= 3\left(C_{11} - C_{12}\right)\varepsilon^2 - 2B_1\varepsilon \end{aligned} \quad (6)$$

To calculate B_1 and C_{11}-C_{12}, we used

$$\begin{aligned} B_1 &= \frac{\partial}{\partial\varepsilon}\frac{E\left(\varepsilon, \alpha = [1,0,0]\right) - E\left(\varepsilon, \alpha = [0,0,1]\right)}{3} \\ C_{11} - C_{12} &= \frac{1}{6}\frac{\partial^2 E\left(\varepsilon, \alpha = [1,0,0]\right)}{\partial\varepsilon^2} \end{aligned} \quad (7)$$

Supplementary Note 2: Phase Field Model

To quantitatively study the permeability and tunability properties of VTIs and elucidate the underlying mechanisms, domain-level phase field modeling and computer simulation are employed to investigate the effects of tunable piezoelectric strain on the permeability and its tunability in ME ferrite/PZT composites under varying voltage and varying MCA energy. In order to treat the strain-mediated domain-level coupling between magnetization and polarization, we adopt the phase field model of ME composites which integrates the phase field models of magnetostrictive materials [2] and ferroelectric materials [3] into one unified model to treat domain processes and grain microstructures in polycrystalline composites of magnetostrictive and ferroelectric phases. In this section, the employed phase field model is briefly described, while more detailed description can be found in previous publication [4].

In the phase field model, the ME composite system can be described by field variables of magnetization **M**(**r**), polarization **P**(**r**), and free charge density ρ(**r**). The total system free energy under externally applied magnetic field $\mathbf{H}^{\mathrm{ex}}$ and electric field $\mathbf{E}^{\mathrm{ex}}$ is [4]:

$$F = \int\left[(1-\eta) f_{\mathrm{M}}\left(R_{ij}M_j\right) + \eta f_{\mathrm{E}}\left(R_{ij}P_j\right) + \beta_{\mathrm{M}}\left|\nabla\mathbf{M}\right|^2 + \beta_{\mathrm{E}}\left|\nabla\mathbf{P}\right|^2 - \mu_0\mathbf{H}^{\mathrm{ex}}\cdot\mathbf{M} - \mathbf{E}^{\mathrm{ex}}\cdot\mathbf{P}\right]d^3r$$
$$+\int\frac{d^3k}{(2\pi)^3}\left[\frac{\mu_0}{2}\left|\mathbf{n}\cdot\mathbf{M}\right|^2 + \frac{1}{2\varepsilon_0}\left|\frac{\rho}{k} - i\mathbf{n}\cdot\mathbf{P}\right|^2 + \frac{1}{2}K_{ijkl}\varepsilon_{ij}\varepsilon_{kl}^*\right] \quad (8)$$

where $f_{\mathrm{M}}(R_{ij}M_j)$ and $f_{\mathrm{E}}(R_{ij}P_j)$ are the local free energy density functions of magnetostrictive and ferroelectric phases, respectively. Both **M**(**r**) and **P**(**r**) are defined in a global coordinate system. The operations $R_{ij}M_j$ and $R_{ij}P_j$ in the functions $f_{\mathrm{M}}(R_{ij}M_j)$ and $f_{\mathrm{E}}(R_{ij}P_j)$ transform **M**(**r**) and **P**(**r**) from the global sample system to the local crystallographic system in each grain, where the grain rotation matrix field R_{ij}(**r**) describes the grain structure and crystallographic orientation of individual grains. The phase field η(**r**) describes magnetostrictive phase by η=0 and ferroelectric phase by η=1. The two-phase morphology of the ME composite is illustrated in Fig. 5(d), where the blue and red color represents the magnetostrictive ferrite and ferroelectric PZT phase, respectively. In the local coordinate system, f_{M}(**M**) is formulated as the MCA energy [5]:

$$f_{\mathrm{M}}(\mathbf{M}) = K_1\left(m_1^2m_2^2 + m_2^2m_3^2 + m_3^2m_1^2\right) + K_2m_1^2m_2^2m_3^2 \quad (9)$$

where **m**=**M**/M is the magnetization direction, and f_{E}(**P**) is formulated by the Landau-Ginzburg-Devonshire (LGD) polynomial energy [6]:

$$
\begin{aligned}
f_{\mathrm{E}}(\mathbf{P}) &= \alpha_1\left(P_1^2+P_2^2+P_3^2\right)+\alpha_{11}\left(P_1^4+P_2^4+P_3^4\right)+\alpha_{12}\left(P_1^2P_2^2+P_2^2P_3^2+P_3^2P_1^2\right) \\
&+\alpha_{111}\left(P_1^6+P_2^6+P_3^6\right)+\alpha_{112}\left[P_1^4\left(P_2^2+P_3^2\right)+P_2^4\left(P_3^2+P_1^2\right)+P_3^4\left(P_1^2+P_2^2\right)\right] \\
&+\alpha_{123}P_1^2P_2^2P_3^2
\end{aligned} \tag{10}
$$

The two gradient terms in Eq. (8) characterize the energy contributions from the magnetization gradient (exchange energy) and polarization gradient, respectively. The **k**-space integral terms characterize the domain configuration-dependent energies of the long-range magnetostatic, electrostatic and elastostatic interactions, where $K_{ijkl}=C_{ijkl}-n_mC_{ijmn}\Omega_{np}C_{klpq}n_q$, $\Omega_{ik}=(C_{ijkl}n_jn_l)^{-1}$, C_{ijkl} is the elastic stiffness tensor, $\mathbf{n}=\mathbf{k}/k$, $\mathbf{M}(\mathbf{k})$, $\mathbf{P}(\mathbf{k})$, $\rho(\mathbf{k})$ and $\boldsymbol{\varepsilon}(\mathbf{k})$ are the Fourier transforms of the respective field variables $\mathbf{M}(\mathbf{r})$, $\mathbf{P}(\mathbf{r})$, $\rho(\mathbf{r})$ and $\boldsymbol{\varepsilon}(\mathbf{r})$. The spontaneous strain $\boldsymbol{\varepsilon}$ is a function of magnetization $\mathbf{M}$ and polarization $\mathbf{P}$, $\varepsilon_{ij}=\lambda_{ijkl}m_km_l+Q_{ijkl}P_kP_l$, where λ_{ijkl} and Q_{ijkl} are magnetostrictive and electrostrictive coefficient tensors, respectively.

The evolution of magnetization $\mathbf{M}(\mathbf{r},t)$ and polarization $\mathbf{P}(\mathbf{r},t)$ are respectively governed by the Landau-Lifshitz-Gilbert equation [6] and the time-dependent Ginzburg-Landau equation [6]:

$$
\frac{\partial\mathbf{M}(\mathbf{r},t)}{\partial t}=\gamma\,\mathbf{M}\times\frac{\delta F}{\delta\mathbf{M}(\mathbf{r},t)}+\alpha\,\mathbf{M}\times\left[\mathbf{M}\times\frac{\delta F}{\delta\mathbf{M}(\mathbf{r},t)}\right] \tag{11}
$$

$$
\frac{\partial\mathbf{P}(\mathbf{r},t)}{\partial t}=-L\frac{\delta F}{\delta\mathbf{P}(\mathbf{r},t)} \tag{12}
$$

where γ is the gyromagnetic ratio and α the damping parameter for magnetization evolution, and L is kinetic coefficient for polarization evolution. The evolution of free charge density field $\rho(\mathbf{r},t)$ is governed by charge conservation and microscopic Ohm's law [7]:

$$
\frac{\partial\rho(\mathbf{r},t)}{\partial t}=-\nabla\cdot\mathbf{j}(\mathbf{r},t) \tag{13}
$$

$$
j_i=\sigma_{ik}E_k \tag{14}
$$

where $\mathbf{j}(\mathbf{r},t)$ is the current density field, $\sigma_{ik}(\mathbf{r})$ describes the electrical conductivity distribution in the ME composite, and $\mathbf{E}(\mathbf{r})$ is the local electric field given by

$$
\mathbf{E}(\mathbf{r})=\mathbf{E}^{\mathrm{ex}}-\frac{1}{\varepsilon_0}\int\frac{d^3k}{(2\pi)^3}\left[\mathbf{n}\cdot\mathbf{P}(\mathbf{k})+i\frac{\rho(\mathbf{k})}{k}\right]\mathbf{n}e^{i\mathbf{k}\cdot\mathbf{r}}\,. \tag{15}
$$

In order to systematically study the MCA effect on permeability and tunability behaviors of the ME ferrite/PZT composite VTI system, various values of MCA constant K_1 are considered in our simulations. Since the saturation magnetostriction constant λ_s and magnetization M_s vary slightly with the small composition of $CoFe_2O_4$ in NiZnCu ferrite alloy according to experimental measurements and first principles calculations, we fixed the values for both λ_s and M_s in all our simulation cases. Therefore, the following material parameters are adopt in our simulations: a series of MCA constant K_1= -10000, -8000, -6000, -4000, -2000, 0, 2000, 4000, 6000, 8000, 10000 J/m^3, saturation magnetization $M_s = 3.5\times10^5$ A/m, saturation magnetostriction constant $\lambda_s = -18\times10^{-6}$ for ferrite material system; and LGD coefficients α_1=-2.67×10^7 m/F, α_{11}=-1.43×10^7 m^5/C^{2}F, α_{12}=1.57×10^7 m^5/C^{2}F, α_{111}=1.34×10^8 m^9/C^{4}F, α_{112}=1.17×10^9 m^9/C^{4}F, α_{123}=-4.77×10^9 m^9/C^{4}F, electrostrictive constants Q_{11}=0.0966 m^4/C^2, Q_{12}=-0.046 m^4/C^2, Q_{44}=0.0819 m^4/C^2 for $Pb(Zr_{0.5}Ti_{0.5})O_3$[6,8].

Supplementary References

1 Fritsch, D. & Ederer, C. First-principles calculation of magnetoelastic coefficients and magnetostriction in the spinel ferrites $CoFe_2O_4$ and $NiFe_2O_4$. *Physical Review B* **86**, 014406 (2012).

2 Huang, Y. Y. & Jin, Y. M. Phase field modeling of magnetization processes in growth twinned Terfenol-D crystals. *Appl. Phys. Lett.* **93**, 142504 (2008).

3 Wang, Y. U. Field-induced inter-ferroelectric phase transformations and domain mechanisms in high-strain piezoelectric materials: insights from phase field modeling and simulation. *J. Mater. Sci.* **44**, 5225-5234 (2009).

4 Ma, F. D., Jin, Y. M., Wang, Y. U., Kampe, S. L. & Dong, S. Phase field modeling and simulation of particulate magnetoelectric composites: Effects of connectivity, conductivity, poling and bias field. *Acta Materialia* **70**, 45-55 (2014).

5 Hubert, A. & Schäfer, R. *Magnetic Domains: The Analysis of Magnetic Microstructures*. first edn, (Springer-Verlag Berlin Heidelberg, 1998).

6 Amin, A., Haun, M. J., Badger, B., McKinstry, H. & Cross, L. E. A phenomenological Gibbs function for the single cell region of the $PbZrO_3$:$PbTiO_3$ solid solution system. *Ferroelectrics* **65**, 107-130 (1985).

7 Jin, Y. M. Phase field modeling of current density distribution and effective electrical conductivity in complex microstructures. *Appl. Phys. Lett.* **103**, 021906 (2013).

8 Haun, M. J., Zhuang, Z. Q., Furman, E., Jang, S. J. & Cross, L. E. Electrostrictive properties of the lead zirconate titanate solid-solution system. *Journal of the American Ceramic Society* **72**, 1140-1144 (1989).
